# Supplementary material for: Advances in Loading Techniques and Quality by Design for Fused Deposition Modeling in Pharmaceutical Production: A Systematic Review
Source: Pharmaceuticals (Basel). 2024 Nov 7;17(11):1496. doi: 10.3390/ph17111496 (PMC11597217; doi:10.3390/ph17111496)
Supplement: Supplementary file 1 [file pharmaceuticals-17-01496-s001.zip › pharmaceuticals-3262207-supplementary.pdf]

Table S1: Summary of the data gathered from publications considered in this systematic review

Drug	Carrier polymer	Loading techniques	Drug amount	3d printer used	Temperature of extrusion	Temperature of printing	Release behaviour	Dosage form	Ref
Nifedipine	Polyvinyl alcohol (PVA)	Soaking	4%	A FlashForge Creator Pro	-	223	Sustained release	Spherical mini-tablets	(1)
	Kollidon VA64 (KVA) and ethylcellulose (EC)	Single screw extruder	30%,40 %,50% and 60%		110-165				
venlafaxine hydrochloride	hydroxypropyl methylcellulose (HPMC) polylactic acid (PLA)	Single screw extruder	25%	Raise 3D Pro2-series	160 °C	185 °C For HPMC 210 °C, for PLA	Sustained release	Intragastric floating tablets	(2)
Deflazacort	poly(e-caprolactone) (PCL) and Eudragit RL100(ERL)	Soaking	0.14%-0.22% for ERL and 0.06%-0.6% for PCL	MakerBot Replicator 2,	-	170 °C ERL and 95°C PCL	Controlled release	Tablets	(3)
paracetamol or caffeine	Polyvinyl alcohol (PVA)	Single screw extruder	5% and 10%	MakerBot Replicator 2X	180 °C	200 °C	Controlled release	Caplets	(4)
Metformin HCl	Polyvinyl alcohol (PVA)	Soaking	0.08% in absolute ethanol and 1.40% in ethanol-water (9:1)	Makerbot experimental 2×	-	205°C	Sustained release	Tablets	(5)
Hydrochlorothiazide	hypromellose acetate succinate (HPMCAS)	Twin-screw extruder	5%	MakerBot Replicator 2X	165	170	Sustained release	Tablets	(6)

Drug	Carrier polymer	Loading techniques	Drug amount	3d printer used	Temperature of extrusion	Temperature of printing	Release behaviour	Dosage form	Ref
Felodipine	Polyvinyl alcohol (PVA)	Single-screw extruder	5% and 15%	MakerBot Replicator 2X	170 and 190°C	165-180	Prolonged release	Tablets	(7)
Isoniazid	Hydroxypropyl Cellulose (HPC)	Single-screw extruder	30%	A MakerBot Replicator 2	100-155	165-195	Sustained release	Tablets	(8)
celecoxib	Polyvinyl Alcohol (PVA)	Single-screw extruder	10%	Prusa i3 MK3S printer	175 °C	205	Sustained release	Tablets	(9)
Pramipexole	combinations of Eudragit EPO and polyethylene oxide	Single-screw extruder	0.7%	MakerBot Inc	120-130	160°C to 175°C	Immediate release	Tablets	(10)
felodipine	Eudragit E PO, Soluplus	Twin-screw extruder	10%	MakerBot Replicator II desktop	100-130	150	Sustained release IN PH6.8 and immediate IN PH 1.2	Discs	(11)
Quinine	Eudragit RS, polycaprolactone (PCL), poly(L-lactide) (PLLA), and ethyl cellulose (EC)	Solvent casting and subsequent hot melt extrusion (HME)	5%	Multirap M420	47-140	53-164	Controlled release	Implants	(12)
5-Fluorouracil	Eudragit® L100-55 and Eudragit® S100	Single-screw extruder	0.54%-2.04%	MakerBot Replicator ® 2X	150 °C, except for one formulation extruded at 165 °C	182 °C	Controlled release	Hollow pH-responsive tablet	(13)
anhydrous caffeine	Poly vinyl alcohol (PVA)	Microwave impregnation	> 0.95%	Ultimaker	-	215	Sustained release	Gastro-retentive capsular devices	(14)

Drug	Carrier polymer	Loading techniques	Drug amount	3d printer used	Temperature of extrusion	Temperature of printing	Release behaviour	Dosage form	Ref
hydrocortisone	Affinisol® 15LV and Kollidon® VA64.	Twin-screw extruder	10% and 20%	Prusa® i3MK3 3D desktop printer	90-140	155-210	Immediate release	Mini-waffle	(15)
paracetamol	hydroxypropyl methylcellulose (HPMC) - Affinisol™ LV15	Twin screw extruder	5 to 50 wt%	STARTT printer kit	150°C - 210°C	190 °C	Sustained release	Tablets	(16)
ketoprofen,	hydroxypropyl methylcellulose (HPMC-K4M), hydroxypropyl methylcellulose acetate succinate (HPMCAS HG), and Hydroxypropyl Cellulose (HPC LF)	Twin-screw extruder	30%	Prusa i3 MK3S kit	150	200 ◦C	Controlled release	Tablets	(17)
Caffeine	Hydroxypropyl-cellulose (HPC), Kollidon VA64 and Kollicoat IR	Twin-screw extruder	5%- 20%	BoltPro 1.75 mm 3D-printer	120°C - 145°C	155-180°C	Immediate release	Tablets	(18)

Drug	Carrier polymer	Loading techniques	Drug amount	3d printer used	Temperature of extrusion	Temperature of printing	Release behaviour	Dosage form	Ref
Acetaminophen	Hydroxypropyl Cellulose (HPC), hydroxypropyl methylcellulose (HPMC), Polyethylene oxide (PEO) and Eudragit RS	Twin-screw extruder	1%	Prusa FDM 3D printer	185 ◦C.	200	Controlled release	Multifunctional core–shell model	(19)
curcumin	Polyvinyl alcohol (PVA)	Soaking	5%	FDM-200W, Ninjabot,	-	210	Delayed release	Tablets	(20)
Atorvastatin calcium and Amlodipine besylate	polyvinyl alcohol (PVA)	Twin-Screw Extruder	2,2.5,5% FOR AMLO 20-30% for atorva	Prusa i3 3D desktop printer	160-180	180-187	Immediate release	Shell-core tablet	(21)
theophylline	Eudragit RL	Twin screw extruder	30%	Prodim XXL printer	140-180	180 C	Sustained release	Tablet	(22)
paracetamol and domperidone	Polyvinyl alcohol (PVA)	Twin screw extruder	para 40% 10-20% for domp	FDM ZMorph® 2.0 S personal fabricator	145-200	165 to 250	Immediate release	Orodispersible tablets	(23)
placebo	hydroxypropyl methylcellulose (HPMC) and ethyl cellulose (EC)	Twin screw extruder		Prusa i3/MK3	190 C	180		Rectangular shapes solid	(24)
ketoprofen	Polyvinyl alcohol (PVA)	Twin screw extruder	20-40-50%	FDM ZMorph® 2.0 S personal fabricator	130°C to 175°C,	175 to 190	Sustained release	Oblong tablets	(25)

Drug	Carrier polymer	Loading techniques	Drug amount	3d printer used	Temperature of extrusion	Temperature of printing	Release behaviour	Dosage form	Ref
Prednisolone	hydroxypropyl methylcellulose (HPMC)	Twin screw extruder	2.5, 5, 10 and 20	Ultimaker 3	150-180	200-220◦C	Not mentioned	Tablets	(26)
naproxen	Kollidon® VA 64	Twin screw extruder	10%, 20%, and 30%	Ultimaker 3 printer	140-160	160	Immediate release	Tablet	(27)
paracetamol, hydrochlorothiazide celecoxib	hydroxypropyl cellulose (HPC EF), Polyvinyl alcohol (PVA) and hydroxypropyl methylcellulose acetate succinate (HPMC-AS LG)	Twin screw extruder	10%, and 30%	Prusa i3 MK3S printer	115-210	140-220	Sustained release	Tablets	(28)
naringenin	hydroxypropyl methylcellulose acetate succinate (HPMCAS), polyvinyl alcohol (PVA), and Eudragit RL PO	Twin screw extruder	over 25%	Voolt FDM 3D printer model Gi3	140-150	180-190	Sustained release	Cylinder shape printlets	(29)
Ramipril	Kollidon VA64 and Kollidon 12PF	Single-screw extruder	3%	MakerBot Replicator 2X Desktop	65-70	90	Immediate release	Tablets	(30)

Drug	Carrier polymer	Loading techniques	Drug amount	3d printer used	Temperature of extrusion	Temperature of printing	Release behaviour	Dosage form	Ref
dipyridamole and theophylline	Eudragit EPO and Eudragit RL	Coordinated 3D printing and liquid dispensing	1.5% of dipyridamole 4% of theophylline	Makerbot Replicator Experimental 2×	-	135-170	Immediate and extended release	Liquid capsules	(31)
paroxetine	hydroxypropyl cellulose (HPC LF)	Single-screw extruder	30%	Delta WASP 20 40 Turbo 2, Wasp	90-120	190-220	Sustained release	Tablets	(32)
Nifedipine	Thermoplastic polyurethane (TPU), polylactic acid (PLA), Polyvinyl alcohol (PVA), and Hydrosupport(96% Polyvinyl alcohol + polyethylene glycol)	Soaking	3% w/w	Voolt FDM 3D printer model Gi3	-	226	Sustained release	Tablets	(33)
Ciprofloxacin HCl	Polypropylene (PP) and Polyvinyl Alcohol (PVA)	Soaking	3 ± 1% w/w for PP and 5 ± 1% w/w for PVA	MakerBot Replicator 2x desktop	-	190-200	Sustained release	Mesh	(34)
ketoprofen	Polyvinyl Alcohol (PVA)	Single-screw extruder	30%	MakerBot Replicator 2X	180	185	Controlled release	Channeled tablet	(35)
indomethacin (IND) and theophylline	polycaprolactone (PCL).	Single-screw extruder	20%,30,40%	Zmorph 3D-printing	100-125	175-190	Prolonged release	Tablets	(36)

Drug	Carrier polymer	Loading techniques	Drug amount	3d printer used	Temperature of extrusion	Temperature of printing	Release behaviour	Dosage form	Ref
Ibuprofen	Ethyl cellulose (EC)	Twin-screw extruder	20%	MakerBot Replicator 2X	80-100	178	Sustained release	Tablets	(37)
Ibuprofen	Hydroxypropyl Methylcellulose Acetate Succinate (HPMCAS)	Twin-screw extruder	20%	Ultimaker S3 printer	130	180	Sustained release	Tablets	(38)
Ketoprofen	Polylactic Acid (PLA)	Supercritical impregnation	9%	-	35,55,75	-	Controlled release	Filament	(39)
mango leaf extract	polylactic acid (PLA)	Supercritical impregnation	3%	ANYCUBIC Mega S model 3D printer	35-55	200	Sustained release	3D-printed disks	(40)
ethanolic mango leaf extract	Polylactic Acid (PLA)	Supercritical impregnation	3%	ANYCUBIC (model MEGA S,	35-55	200	Sustained release	Biomedical devices	(41)
sodium warfarin	Eudragit EPO	Twin-screw extruder	1%	MakerBot Replicator 2X	90-100	135	Immediate release	Tablet	(42)
indomethacin	poly(ε-caprolactone) (PCL)	Twin-screw extruder	5%, 15%, and 30%	MakerBot Replicator 2 desktop	100	100	Controlled release	Implants	(43)
Fenofibrate	Hydroxypropyl Methylcellulose Acetate Succinate (HPMC AS LG)	Twin-screw extruder	20%	Batch 3D printer	90-130	140	Sustained release	Tablets	(44)

Drug	Carrier polymer	Loading techniques	Drug amount	3d printer used	Temperature of extrusion	Temperature of printing	Release behaviour	Dosage form	Ref
glibenclamide	polylactic acid (PLA)	Soaking	0.6%	Zmorph VX 3D-printing system	-	180-200	Sustained release	Tablets	(45)

1. Ayyoubi S, Cerda JR, Fernández-García R, Knief P, Lalatsa A, Healy AM, et al. 3D printed spherical mini-tablets: Geometry versus composition effects in controlling dissolution from personalised solid dosage forms. International journal of pharmaceutics. 2021;597:120336.
2. Zhao X, Wei W, Niu R, Li Q, Hu C, Jiang S. 3D Printed Intragastric Floating and Sustained-Release Tablets with Air Chambers. Journal of pharmaceutical sciences. 2022;111(1):116-23.
3. Beck RCR, Chaves PS, Goyanes A, Vukosavljevic B, Buanz A, Windbergs M, et al. 3D printed tablets loaded with polymeric nanocapsules: An innovative approach to produce customized drug delivery systems. International journal of pharmaceutics. 2017;528(1-2):268-79.
4. Goyanes A, Wang J, Buanz A, Martínez-Pacheco R, Telford R, Gaisford S, et al. 3D Printing of Medicines: Engineering Novel Oral Devices with Unique Design and Drug Release Characteristics. Pharmaceutics. 2015;12(11):4077-84.
5. Ibrahim M, Barnes M, McMillin R, Cook DW, Smith S, Halquist M, et al. 3D Printing of Metformin HCl PVA Tablets by Fused Deposition Modeling: Drug Loading, Tablet Design, and Dissolution Studies. AAPS PharmSciTech. 2019;20(5):195.
6. Oladeji S, Mohylyuk V, Jones DS, Andrews GP. 3D printing of pharmaceutical oral solid dosage forms by fused deposition: The enhancement of printability using plasticised HPMCAS. Pharmaceutical development and technology. 2022;616:121553.
7. Iovanov RI, Porfire AS, Crisan AG, Dobre AA, Iurian SM, Rus LM, et al. 3D PRINTING OF PROLONGED-RELEASE ORAL SOLID DOSAGE FORMS CONTAINING FELODIPINE. Farmacia. 2023;71(3):480-90.
8. Öblom H, Zhang JX, Pimparade M, Speer I, Preis M, Repka M, et al. 3D-Printed Isoniazid Tablets for the Treatment and Prevention of TuberculosisPersonalized Dosing and Drug Release. AAPS PharmSciTech. 2019;20(2).
9. Katsiotis CS, Åhlén M, Stromme M, Welch K. 3D-Printed Mesoporous Carrier System for Delivery of Poorly Soluble Drugs. Pharmaceutics. 2021;13(7).
10. Gültekin HE, Tort S, Acartürk F. An Effective Technology for the Development of Immediate Release Solid Dosage Forms Containing Low-Dose Drug: Fused Deposition Modeling 3D Printing. Pharmaceutical research. 2019;36(9):128.
11. Alhijjaj M, Belton P, Qi S. An investigation into the use of polymer blends to improve the printability of and regulate drug release from pharmaceutical solid dispersions prepared via fused deposition modeling (FDM) 3D printing. European Journal of Pharmaceutics and Biopharmaceutics. 2016;108:111-25.
12. Kempin W, Franz C, Koster LC, Schneider F, Bogdahn M, Weitschies W, et al. Assessment of different polymers and drug loads for fused deposition modeling of drug loaded implants. European journal of pharmaceutics and biopharmaceutics : official journal of Arbeitsgemeinschaft fur Pharmazeutische Verfahrenstechnik eV. 2017;115:84-93.

13. Gioumouxouzis CI, Chatzitaki AT, Karavasili C, Katsamenis OL, Tzetzis D, Mystiridou E, et al. Controlled Release of 5-Fluorouracil from Alginate Beads Encapsulated in 3D Printed pH-Responsive Solid Dosage Forms. AAPS PharmSciTech. 2018;19(8):3362-75.
14. Saviano M, Bowles BJ, Penny MR, Ishaq A, Muwaffak Z, Falcone G, et al. Development and analysis of a novel loading technique for FDM 3D printed systems: Microwave-assisted impregnation of gastro-retentive PVA capsular devices. International journal of pharmaceutics. 2022;613.
15. Parulski C, Bya LA, Goebel J, Servais AC, Lechanteur A, Evrard B. Development of 3D printed mini-waffle shapes containing hydrocortisone for children's personalized medicine. International journal of pharmaceutics. 2023;642:123131.
16. Prasad E, Islam MT, Goodwin DJ, Megarry AJ, Halbert GW, Florence AJ, et al. Development of a hot-melt extrusion (HME) process to produce drug loaded Affinisol™ 15LV filaments for fused filament fabrication (FFF) 3D printing. Additive Manufacturing. 2019;29.
17. Hu ZQ, Xu PC, Zhang JX, Bandari S, Repka MA. Development of controlled release oral dosages by density gradient modification via three-dimensional (3D) printing and hot-melt extrusion (HME) technology. Journal of Drug Delivery Science and Technology. 2022;71.
18. Fanous M, Gold S, Hirsch S, Ogorka J, Imanidis G. Development of immediate release (IR) 3D-printed oral dosage forms with focus on industrial relevance. European Journal of Pharmaceutical Sciences. 2020;155.
19. Zhang ZR, Feng S, Almotairy A, Bandari S, Repka MA. Development of multifunctional drug delivery system via hot-melt extrusion paired with fused deposition modeling 3D printing techniques. European Journal of Pharmaceutics and Biopharmaceutics. 2023;183:102-11.
20. Tagami T, Kuwata E, Sakai N, Ozeki T. Drug Incorporation into Polymer Filament Using Simple Soaking Method for Tablet Preparation Using Fused Deposition Modeling. Biological & Pharmaceutical Bulletin. 2019;42(10):1753-60.
21. Alzahrani A, Narala S, Youssef AAA, Nyavanandi D, Bandari S, Mandati P, et al. Fabrication of a shell-core fixed-dose combination tablet using fused deposition modeling 3D printing. European Journal of Pharmaceutics and Biopharmaceutics. 2022;177:211-23.
22. Korte C, Quodbach J. Formulation development and process analysis of drug-loaded filaments manufactured via hot-melt extrusion for 3D-printing of medicines. Pharmaceutics. 2018;23(10):1117-27.
23. Tranová T, Pyteraf J, Kurek M, Jamróz W, Brniak W, Spalovská D, et al. Fused Deposition Modeling as a Possible Approach for the Preparation of Orodispersible Tablets. Pharmaceuticals. 2022;15(1).
24. Ponsar H, Wiedey R, Quodbach J. Hot-Melt Extrusion Process Fluctuations and Their Impact on Critical Quality Attributes of Filaments and 3D-Printed Dosage Forms. Pharmaceutics. 2020;12(6).
25. Pyteraf J, Jamróz W, Kurek M, Szafraniec-Szczesny J, Kramarczyk D, Jurkiewicz K, et al. How to Obtain the Maximum Properties Flexibility of 3D Printed Ketoprofen Tablets Using Only One Drug-Loaded Filament? Molecules (Basel, Switzerland). 2021;26(11).
26. Larsen BS, Kissi E, Nogueira LP, Genina N, Tho I. Impact of drug load and polymer molecular weight on the 3D microstructure of printed tablets. European journal of pharmaceutical sciences : official journal of the European Federation for Pharmaceutical Sciences. 2024;192:106619.
27. Kissi EO, Nilsson R, Nogueira LP. Influence of Drug Load on the Printability and Solid-State Properties of 3D-Printed Naproxen-Based Amorphous Solid Dispersion. 2021;26(15).
28. Macedo J, Vanhoorne V, Vervaet C, Pinto JF. Influence of formulation variables on the processability and properties of tablets manufactured by fused deposition modelling. International journal of pharmaceutics. 2023;637:122854.

29. Pires FQ, Gross IP, Sa-Barreto LL, Gratieri T, Gelfuso GM, Bao SN, et al. In-situ formation of nanoparticles from drug-loaded 3D polymeric matrices. European journal of pharmaceutical sciences : official journal of the European Federation for Pharmaceutical Sciences. 2023;188:106517.
30. Kollamaram G, Croker DM, Walker GM, Goyanes A, Basit AW, Gaisford S. Low temperature fused deposition modeling (FDM) 3D printing of thermolabile drugs. International journal of pharmaceutics. 2018;545(1-2):144-52.
31. Okwuosa TC, Soares C, Gollwitzer V, Habashy R, Timmins P, Alhnan MA. On demand manufacturing of patient-specific liquid capsules via coordinated 3D printing and liquid dispensing. European Journal of Pharmaceutical Sciences. 2018;118:134-43.
32. Figueiredo S, Fernandes AI, Carvalho FG, Pinto JF. Performance and paroxetine stability in tablets manufactured by fused deposition modelling-based 3D printing. Journal of Pharmacy and Pharmacology. 2022;74(1):67-76.
33. Cerda JR, Arifi T, Ayyoubi S, Knief P, Ballesteros MP, Keeble W, et al. Personalised 3D Printed Medicines: Optimising Material Properties for Successful Passive Diffusion Loading of Filaments for Fused Deposition Modelling of Solid Dosage Forms. Pharmaceutics. 2020;12(4).
34. Qamar N, Abbas N, Irfan M, Hussain A, Arshad MS, Latif S, et al. Personalized 3D printed ciprofloxacin impregnated meshes for the management of hernia. Journal of Drug Delivery Science and Technology. 2019;53.
35. Crisan AG, Porfire A, Ambrus R, Katona G, Rus LM, Porav AS, et al. Polyvinyl Alcohol-Based 3D Printed Tablets: Novel Insight into the Influence of Polymer Particle Size on Filament Preparation and Drug Release Performance. Pharmaceuticals. 2021;14(5).
36. Viidik L, Vesala J, Laitinen R, Korhonen O, Ketolainen J, Aruväli J, et al. Preparation and characterization of hot-melt extruded polycaprolactone-based filaments intended for 3D-printing of tablets. European journal of pharmaceutical sciences : official journal of the European Federation for Pharmaceutical Sciences. 2021;158:105619.
37. Shi K, Salvage JP, Maniruzzaman M, Nokhodchi A. Role of release modifiers to modulate drug release from fused deposition modelling (FDM) 3D printed tablets. International journal of pharmaceutics. 2021;597:120315.
38. Zhang J, Thakkar R, Zhang Y, Maniruzzaman M. Structure-function correlation and personalized 3D printed tablets using a quality by design (QbD) approach. International journal of pharmaceutics. 2020;590:119945.
39. Naranjo LV, Bastante CC, Cardoso LC, Serrano CM, Fernández E. Supercritical Impregnation of Ketoprofen into Polylactic Acid for Biomedical Application: Analysis and Modeling of the Release Kinetic. Polymers. 2021;13(12).
40. Grosso P, Cejudo C, Sánchez-Gomar I, Durán-Ruiz MC, Moreno-Luna R, Casas L, et al. Supercritical Impregnation of Mango Leaf Extract into PLA 3D-Printed Devices and Evaluation of Their Biocompatibility with Endothelial Cell Cultures. Polymers. 2022;14(13).
41. Rosales JM, Cejudo C, Verano L, Casas L, Mantell C, de la Ossa EJM. Supercritical Impregnation of PLA Filaments with Mango Leaf Extract to Manufacture Functionalized Biomedical Devices by 3D Printing. Polymers. 2021;13(13).
42. Arafat B, Qinna N, Cieszynska M, Forbes RT, Alhnan MA. Tailored on demand anti-coagulant dosing: An in vitro and in vivo evaluation of 3D printed purpose-designed oral dosage forms. Pharmaceutics. 2018;128:282-9.
43. Tarasiuk J, Wroński S, Paluch M, Jachowicz R, Holländer J, Genina N, et al. Three-Dimensional Printed PCL-Based Implantable Prototypes of Medical Devices for Controlled Drug Delivery. Molecules (Basel, Switzerland). 2016;105(9):2665-76.
44. Kulkarni VR, Chakka J, Alkadi F, Maniruzzaman M. Veering to a Continuous Platform of Fused Deposition Modeling Based 3D Printing for Pharmaceutical Dosage Forms: Understanding the Effect of Layer Orientation on Formulation Performance. Pharmaceutics. 2023;15(5).

45. Kukkonen J, Ervasti T, Laitinen R. Production and characterization of glibenclamide incorporated PLA filaments for 3D printing by fused deposition modeling. Journal of Drug Delivery Science and Technology. 2022;77:103843.

Table S2: Data maxtrix after data curation for statistical analysis

Carrier polymer	Loading technique	Max. drug amount (%)	Max. pretreat. Temp (°C)	Printing temp. (°C)	Release time (h)
Ethyl cellulose (EC)	Extrusion	20	100	178	24
Eudragit EPO	Extrusion	1	100	135	0,5
Eudragit RL	Extrusion	30	180	180	24
Hydroxypropyl Cellulose (HPC)	Extrusion	30	120	220	12
Hydroxypropyl Cellulose (HPC)	Extrusion	30	155	195	14
Hydroxypropyl methylcellulose (HPMC)	Extrusion	25	160	185	24
Hydroxypropyl Methylcellulose Acetate Succinate (HPMC AS)	Extrusion	20	130	140	8
Hydroxypropyl Methylcellulose Acetate Succinate (HPMC AS)	Extrusion	20	130	180	6,6
Hydroxypropyl Methylcellulose Acetate Succinate (HPMC AS)	Extrusion	25	150	190	24
Hydroxypropyl Methylcellulose Acetate Succinate (HPMC AS)	Extrusion	5	165	170	3
Kollidon® VA 64	Extrusion	30	160	160	0,75
poly(ε-caprolactone) (PCL)	Extrusion	30	100	100	720
poly(ε-caprolactone) (PCL)	Extrusion	40	125	190	24
Polylactic acid (PLA)	Extrusion	25	160	210	24
Polymer mixture	Extrusion	20	140	210	0,5
Polymer mixture	Extrusion	0,7	130	175	0,5
Polymer mixture	Extrusion	10	130	150	0,5
Polymer mixture	Extrusion	5	140	164	4248
Polymer mixture	Extrusion	2,04	150	182	9
Polymer mixture	Extrusion	30	210	220	6
Polymer mixture	Extrusion	1	185	200	12

Polymer mixture	Extrusion	50	210	190	6
Polymer mixture	Extrusion	30	150	200	24
Polymer mixture	Extrusion	20	145	180	0,5
Polymer mixture	Extrusion	60	165	223	6
Polymer mixture	Extrusion	3	70	90	30
Polyvinyl alcohol (PVA)	Extrusion	10	180	200	6
Polyvinyl alcohol (PVA)	Extrusion	30	180	185	8
Polyvinyl alcohol (PVA)	Extrusion	10	175	205	24
Polyvinyl alcohol (PVA)	Extrusion	40	145	250	0,25
Polyvinyl alcohol (PVA)	Extrusion	50	175	190	5
Polyvinyl alcohol (PVA)	Extrusion	15	190	180	5
polyvinyl alcohol (PVA)	Extrusion	30	180	187	0,5
Eudragit RL100	Impregnation	0,22	25	170	24
poly(ε-caprolactone) (PCL)	Impregnation	0,6	25	95	24
Polylactic acid (PLA)	Impregnation	0,6	25	200	24
Polylactic acid (PLA)	Impregnation	3	55	200	6
Polylactic Acid (PLA)	Impregnation	3	55	200	600
Polylactic Acid (PLA)	Impregnation	9	75	200	24
Polymer mixture	Impregnation	4	25	170	16
Polymer mixture	Impregnation	1,5	25	170	16
Polymer mixture	Impregnation	3	25	226	24
Polypropylene (PP)	Impregnation	5	25	200	12
Polyvinyl alcohol (PVA)	Impregnation	0,95	25	215	6
Polyvinyl alcohol (PVA)	Impregnation	3	25	200	12
Polyvinyl alcohol (PVA)	Impregnation	1,4	25	205	3
Polyvinyl alcohol (PVA)	Impregnation	5	25	210	24
